# *In Situ* Proximity Ligation Assay Reveals Co-Localization of Alpha-Synuclein and SNARE Proteins in Murine Primary Neurons

**DOI:** 10.3389/fneur.2018.00180

**Published:** 2018-03-22

**Authors:** Leire Almandoz-Gil, Emma Persson, Veronica Lindström, Martin Ingelsson, Anna Erlandsson, Joakim Bergström

**Affiliations:** Department of Public Health and Caring Sciences, Molecular Geriatrics, Uppsala University, Uppsala, Sweden

**Keywords:** alpha-synuclein, SNARE, VAMP-2, SNAP-25, syntaxin-1, proximity ligation assay, primary neurons

## Abstract

The aggregation of alpha-synuclein (αSyn) is the pathological hallmark of Parkinson’s disease, dementia with Lewy bodies and related neurological disorders. However, the physiological function of the protein and how this function relates to its pathological effects remain poorly understood. One of the proposed roles of αSyn is to promote the soluble *N*-ethylmaleimide-sensitive factor attachment protein receptor (SNARE) complex assembly by binding to VAMP-2. The objective of this study was to visualize the co-localization between αSyn and the SNARE proteins (VAMP-2, SNAP-25, and syntaxin-1) for the first time using *in situ* proximity ligation assay (PLA). Cortical primary neurons were cultured from either non-transgenic or transgenic mice expressing human αSyn with the A30P mutation under the Thy-1 promoter. With an antibody recognizing both mouse and human αSyn, a PLA signal indicating close proximity between αSyn and the three SNARE proteins was observed both in the soma and throughout the processes. No differences in the extent of PLA signals were seen between non-transgenic and transgenic neurons. With an antibody specific against human αSyn, the PLA signal was mostly located to the soma and was only present in a few cells. Taken together, *in situ* PLA is a method that can be used to investigate the co-localization of αSyn and the SNARE proteins in primary neuronal cultures.

## Introduction

Alpha-synuclein (αSyn) is a presynaptic protein implicated in the pathology of Parkinson’s disease (PD), dementia with Lewy bodies (DLB) and multiple system atrophy. In its native state αSyn is an unfolded monomer, but under certain circumstances it can adopt a partly folded conformation and start an aggregation cascade, resulting in oligomers of increasing sizes and finally insoluble fibrils (1). The resulting inclusions, known as Lewy bodies and Lewy neurites, are the pathological hallmark of PD and related diseases (2). The presence of these inclusions is not enough to explain the neurodegeneration in the actual disorders, as the amount of Lewy bodies does not correlate with disease severity (3, 4). It has been suggested instead that smaller αSyn aggregates can cause synaptic dysfunction and loss of synapses. For example, up to 90% of all αSyn aggregates have been described to be located at the synapses in DLB brains with an associated loss of presynaptic proteins (5).

The physiological function of αSyn has remained unclear, but due to its presynaptic localization and interactions with membrane lipids (6) it has been hypothesized that it might play a role in neurotransmitter release. Recently, αSyn has been reported to chaperone the assembly of the soluble *N*-ethylmaleimide-sensitive factor attachment protein receptor (SNARE) complex by interacting with VAMP-2 (7). The SNARE proteins are critical for neurotransmitter release, as their assembly into a complex promotes the fusion of synaptic vesicles to the presynaptic membrane. They are classified in two groups: vesicle-SNAREs (v-SNAREs), which are bound to synaptic vesicles, i.e., VAMP-2, and the target-SNAREs (t-SNAREs), which are bound to the presynaptic membrane, i.e., SNAP-25 and syntaxin-1. When v-SNAREs and t-SNAREs assemble into a complex, the membranes fuse (8). Experimental evidence suggests that alpha-synuclein directly binds the N-terminus of VAMP-2 (1–28) with its C-terminus (96–140) (9).

It is unknown if the interaction between αSyn and the SNARE proteins is affected in PD and other α-synucleinopathies. Previous studies on mouse models for such disorders have shown that a 1–120 truncated αSyn can cause the redistribution of VAMP-2, SNAP-25, and syntaxin-1 in the synapse (10). One of the most widely studied αSyn transgenic (tg) models expresses human αSyn (h-αSyn) containing the point mutation A30P, which has been found to cause PD in humans (11). The tg A30P αSyn mice present PD-like motor symptoms at around 8 months of age and there are abundant αSyn aggregates found throughout the brainstem and midbrain (12–15). The motor symptoms have been associated with an increase in soluble αSyn protofibrils in the spinal cord (16). However, it is unknown whether the interaction between αSyn and the SNARE proteins is affected in these mice.

Interaction between two proteins in either cells or tissues can be visualized with the proximity ligation assay (PLA) (17). With this technique, the protein partners are targeted with two primary antibodies raised in different species and a pair of oligonucleotide labeled secondary antibodies (PLA probes). If the proteins of interest are located in close proximity to each other, two circularization oligonucleotides will be hybridized to the PLA probes and then ligated together, forming a circular DNA strand which will be the template for a rolling circle amplification step. Fluorescently labeled oligonucleotides will then be added and hybridized with the concatemeric construct.

In this study, we used PLA to investigate the molecular interaction between αSyn and the SNARE proteins in cortical primary neurons from non-tg and tg (Thy-1)-h[A30P] αSyn mice.

## Materials and Methods

### Animals

All animal experiments were approved by the animal ethics committee of Uppsala, Sweden (C75/13, C92/14). The use and care of the animals were conducted in accordance with the EU Directive 2010/63/EU for animal experiments. C57BL/6 and Tg (Thy-1)-h[A30P] αSyn mice pregnant females were used for the extraction of E14 embryos. Non-transgenic (C57BL/6) mice were obtained from Jackson laboratory (Bar Harbor, ME, USA).

### Cortical Primary Neuron Cultures

Cortices from E14 embryos were dissected in Hank’s buffered salt solution supplemented with 100 U/ml penicillin, 100 µg/ml streptomycin, and 8 mM HEPES buffer (Thermo Fisher Scientific, Waltham, MA, USA). Approximately 90,000 cells/ml were plated on poly-l-ornithine (Sigma-Aldrich, St. Louis, MO, USA) and laminin coated (Thermo Fisher Scientific) cover slips. Cells were grown in neurobasal medium supplemented with B27, 100 U/ml penicillin, 100 µg/ml streptomycin, and l-glutamine 2 mM (Thermo Fisher Scientific). The cells were maintained at 37°C, 5% CO_2_, until they were fixated with paraformaldehyde 4% 12 days later.

### Cell Lysis

The cells were washed with phosphate-buffered saline (PBS) containing a protease inhibitor cocktail tablet (PIC, cOmplete, EDTA-free, Roche, Basel, Switzerland) and scraped. After centrifugation at 2,000 × *g* for 5 min, the lysis was performed incubating the cells in PBS with PIC and 1% Triton X-100 (Sigma-Aldrich) for 5 min. After centrifuging at 16,000 × *g* for 5 min, the supernatant was saved.

### Sandwich Enzyme-Linked Immunosorbent Assay

A 96-well high-binding polystyrene plate (Corning Inc., Corning, NY, USA) was coated with 50 ng/well of either mouse monoclonal clone 42/alpha-synuclein antibody (BD Biosciences, San Jose, CA, USA) for total (i.e., m-αSyn and h-αSyn) αSyn detection or mouse monoclonal 4B12 anti-αSyn antibody (Eurogentec, Osaka, Japan) for detection of h-αSyn and was incubated at 4°C overnight. After blocking the plate for 2 h with PBS supplemented with 1% bovine serum albumin (Sigma-Aldrich), the primary neuron lysates were incubated for 2 h at room temperature, together with serial dilutions of recombinant monomeric αSyn as a standard. Next the detection antibody FL-140 (Santa Cruz Biotechnologies, Santa Cruz, CA, USA) was incubated for 1 h at room temperature at 1 µg/ml, followed by 1 h incubation of goat-anti-rabbit-HRP secondary antibody at 1:10,000 at room temperature. The reaction was developed with K-Blue Aqueous TMB substrate (Neogen Corporation, Lexington, KY, USA) and 1 M sulfuric acid (Sigma-Aldrich). Between every step 5× washes were performed with washing buffer (6.5 mM sodium dihydrogen phosphate monohydrate, 43.5 mM di-sodium hydrogen phosphate dihydrate, 0.3 M sodium chloride, and 0.1% Tween-20) in a HydroSpeed microplate washer (Tecan, Männedorf, Switzerland). The absorbance was measured at 450 nm using an Infinite M200 Pro microplate reader (Tecan). The reactions were performed in duplicates and their signal was averaged. The blank signal was deducted from the sample signal.

### Antibodies

The following antibodies were used for the immunofluorescence and PLA experiments, with the same concentration for both techniques: mouse monoclonal mAb1338 recognizing both endogenous mouse αSyn (m-αSyn) and human αSyn (h-αSyn) at 4 µg/ml (R&D Systems, Minneapolis, MN, USA), mouse monoclonal Syn 204 against h-αSyn at 4 µg/ml (Santa Cruz Biotechnology), rabbit monoclonal EPR12790 against VAMP-2 at 2 µg/ml (Abcam, Cambridge, UK), rabbit monoclonal EP3274 against SNAP-25 at 4 µg/ml (Abcam), rabbit polyclonal against syntaxin-1 at 1:1,000 (ABR-Affinity Bioreagents, Golden, CO, USA), rabbit polyclonal AB5622 against microtubule associate protein MAP2 at 1:200 (Merck Millipore, Burlington, MA, USA), and rabbit monoclonal C39A9 against nucleoporin NUP98 at 1:50 (Cell Signaling Technologies, Danvers, MA, USA).

### Immunofluorescence

Fixed cells were permeabilized and blocked with PBS containing 0.1% Triton X-100 and 5% normal goat serum for 30 min at room temperature. The cells were incubated with primary antibodies for 1 h at room temperature in PBS with normal goat serum 0.5%. After three PBS washes, the cells were incubated with the secondary antibodies (goat anti-rabbit Alexa 488 or goat anti-mouse Alexa 594, Thermo Fisher Scientific) at 2 μg/ml in PBS with normal goat serum 0.5% for 1 h at room temperature. After three PBS washes, the cover slips were mounted with Vectashield hard set mounting medium with DAPI (Vector Laboratories, Burlingame, CA, USA).

### “*In situ*” PLA

The *in situ* PLA was performed on fixed primary neurons with DuoLink PLA technology probes and reagents (Sigma-Aldrich), and following the manufacturers protocol. First the neurons were permeabilized with PBS + Triton X-100 0.4% for 10 min. After two PBS washes, the cells were incubated with blocking solution for 30 min at 37°C and then with the primary antibodies for 1 h at room temperature. Every experiment was performed with a pair of antibodies of different species. The cover slips were washed twice for 5 min with buffer A, followed by incubation with the PLA probes (secondary antibodies against two different species bound to two oligonucleotides: anti-mouse MINUS and anti-rabbit PLUS) in antibody diluent for 60 min at 37°C. After two washes of 5 min with buffer A, the ligation step was performed with ligase diluted in ligation stock for 30 min at 37°C. In the ligation step, the two oligonucleotides in the PLA probes are hybridized to the circularization oligonucleotides. The cells were washed with buffer A twice for 2 min before incubation for 100 min with amplification stock solution at 37°C. The amplification stock solution contains polymerase for the rolling circle amplification step and oligonucleotides labeled with fluorophores, which will bind to the product of the rolling circle amplification and thus allow detection. After two washes of 10 min with buffer B, the cells were incubated with FITC-conjugated phalloidin (Sigma-Aldrich) at 1.25 µg/ml. Finally, the cover slips were washed with PBS and mounted with Duolink *in situ* mounting medium containing DAPI. For every antibody, a negative control experiment was performed where only one antibody was incubated with the PLA probes. The experiments were performed three times on non-tg neurons and twice on tg neurons. Each experiment was performed with neurons originated from embryos of different mothers.

### Microscopy and Image Analysis

Immunofluorescence staining was imaged with a Zeiss Axio Observer Z1 (Zeiss, Oberkochen, Germany). Quantification of h-αSyn positive cells was performed in 10 images of non-tg cells and 20 images of tg cells.

Proximity ligation assay imaging was performed with a confocal laser scanning microscope (LSM700, Zeiss). z-Stacks were captured with sections spanning entire cells. Zeiss software Zen 2.3 Blue edition was used to obtain maximum intensity projections and cross-sections of the confocal images. Quantification of PLA signal was performed on z-stack images taken systematically with a Zeiss Axio Observer Z1 microscope. Background signal was reduced by deconvolution using the Huygen software. Live cells with non-condensed nuclei were manually counted as number of neurons per image. The soma was manually outlined for each cell and number of puncta per soma was counted with ImageJ 3D Objects Counter. The number of puncta in the processes was calculated by counting total number of puncta per image and subtracting number of puncta in the soma. The number of puncta was normalized by number of cells to obtain mean PLA puncta/cell. The threshold was set automatically using ImageJ 3D Objects Counter for each image and kept constant as the puncta in the soma were measured separately. Ten z-stack images were quantified per staining. The microscope settings were kept constant for all images to enable direct comparison. The quantification was performed on one set of experiments, when all stainings were performed at the same time. A sampling of the images is included as Figure S1 in Supplementary Material, with examples of the final thresholded images that were quantified. Quantification of cells with positive PLA signal between h-αSyn and SNARE proteins was performed manually in 10 images of tg cells.

### Statistical Analysis

The data were analyzed using GraphPad Prism. Two-tailed Student’s *t*-test was used to compare the amount of alpha-synuclein in cell lysates. One-way ANOVA followed by Bonferroni *post hoc* test was performed to compare the PLA puncta in soma/processes of non-tg and tg (Thy-1)-h[A30P] primary neurons.

## Results

The aim of this study was to visualize the co-localization between αSyn and SNARE proteins in non-transgenic primary neurons and primary neurons overexpressing A30P h-αSyn using PLA. First, we cultured cortical primary neurons of non-tg mouse embryos to observe the localization of mouse αSyn (m-αSyn) and the SNARE proteins, using conventional immunofluorescence. In addition to characterizing the expression pattern of the proteins of interest, it ensured that the antibodies were suitable candidates for PLA.

**Figure 1:**
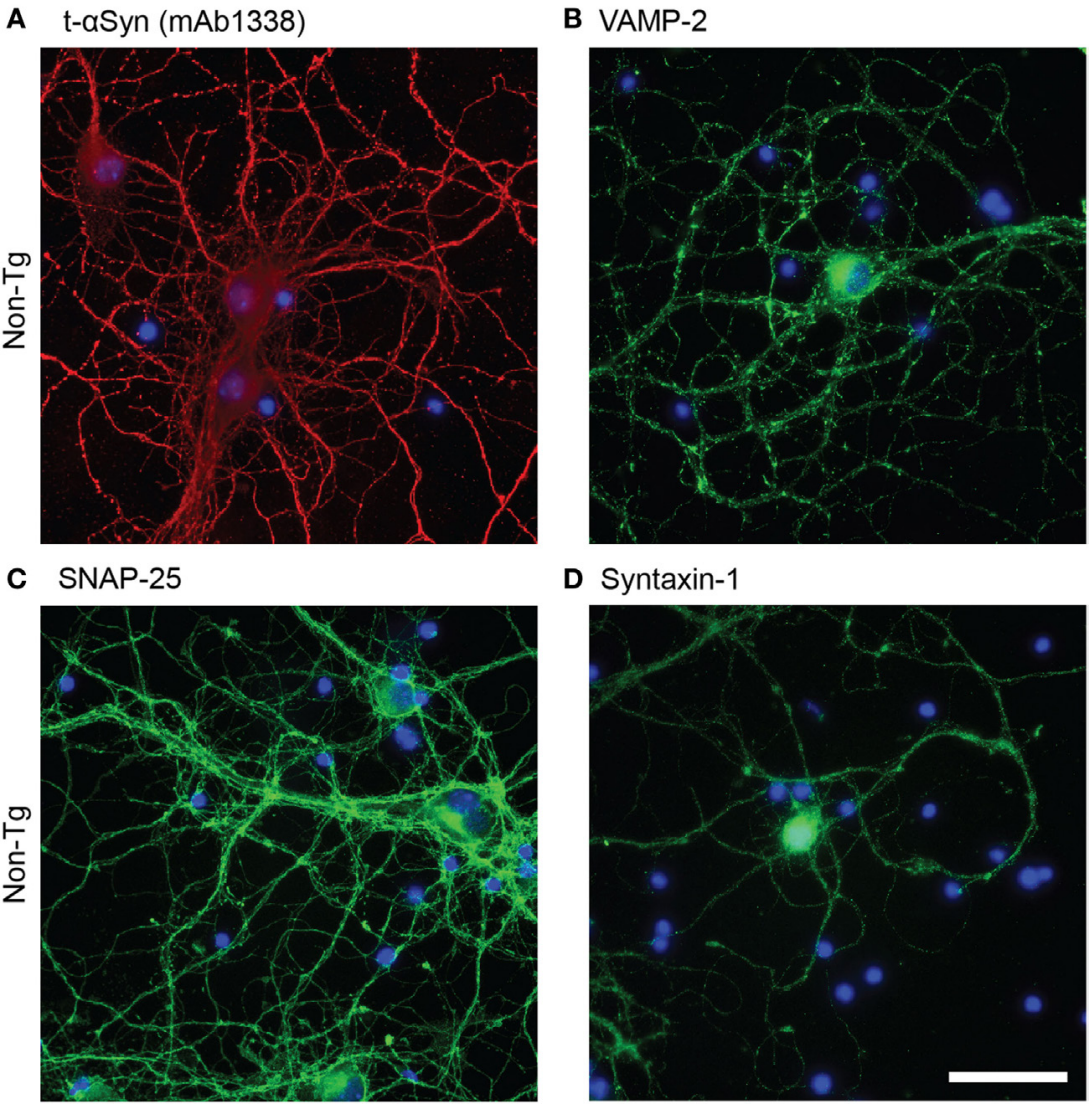
Representative images of immunofluorescence staining of non-tg cortical primary neurons (12 DIV) using antibodies against alpha-synuclein (αSyn) [mAb1338, red, **(A)**], VAMP-2 [(green), **(B)**], SNAP-25 [(green), **(C)**], and syntaxin-1 [(green), **(D)**]. DAPI in blue. Scale bar 50 µm.

Antibody mAb1338 recognizes both m-αSyn and h-αSyn and can thus be used to detect total alpha-synuclein (t-αSyn). The αSyn staining was observed throughout the cell body and processes (Figure 1A). Similarly, the SNARE proteins (VAMP-2, SNAP-25, and syntaxin-1) were abundantly expressed (Figure 1B-D), particularly in cell processes, where a more dotted pattern could be observed; presumably due to the presence in the synaptic boutons.

**Figure 2:**
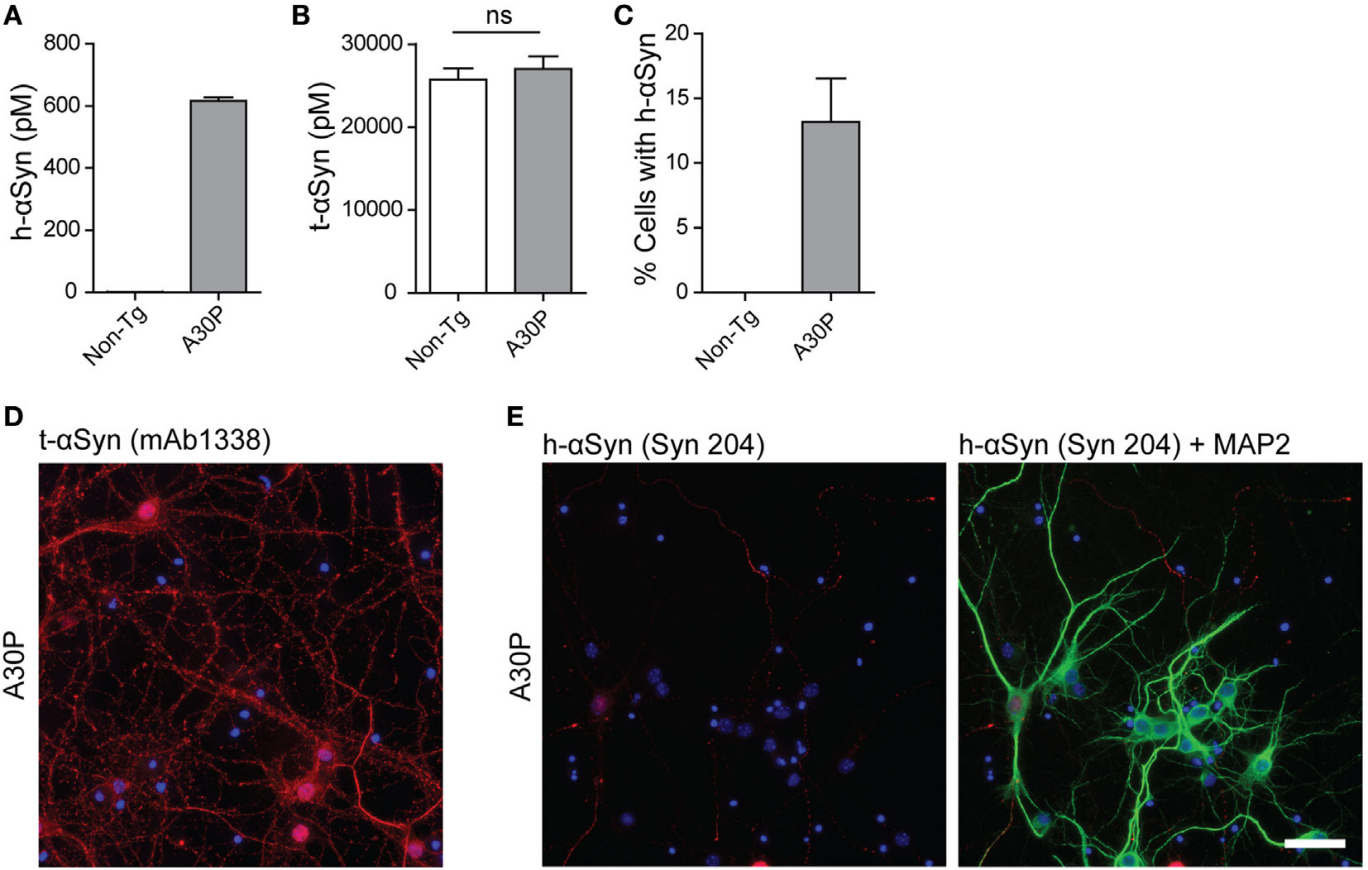
Characterization of primary neurons from tg A30P mice. Levels of h-alpha-synuclein (αSyn) in lysates of non-tg and A30P neuronal cultures were measured by ELISA, using the h-αSyn specific antibody 4B12 as a capture antibody **(A)**. Levels of total (t)-αSyn (m-αSyn + h-αSyn) in lysates of non-tg and A30P neuronal cultures were measured by ELISA, using clone 42 as a capture antibody. Two-tailed Student’s *t*-test, non-significant (ns), error bars represent the SEM **(B)**. Quantification of h-αSyn positive non-tg and A30P neurons, observed by immunofluorescence with h-αSyn specific antibody Syn 204 **(C)**. Representative images of immunofluorescence of tg A30P neurons with t-αSyn mAb1338 **(D)**. Representative images of immunofluorescence with h-αSyn specific antibody Syn 204 of A30P cortical neurons (red). MAP2 neuronal marker (green) and DAPI (blue) **(E)**. Scale bar 50 µm.

We next cultured cortical primary neurons from tg (Thy-1)-h[A30P] αSyn mouse embryos and analyzed the expression and localization of m-αSyn and h-αSyn by ELISA on cell lysates and immunofluorescence (Figure 2). Human-αSyn was detected in lysates from A30P primary neurons, at a concentration of 600 pM and, as expected, not at all in non-tg primary neurons (Figure 2A). The levels of t-αSyn (m-αSyn in the case of non-tg neurons and both m-αSyn and h-αSyn in tg neurons) were found to be similar in both types of primary neurons, with h-αSyn constituting approximately 2% of all αSyn detected in A30P primary neurons (Figure 2B). We performed immunofluorescence of the cultured A30P neurons with the h-αSyn-specific antibody Syn 204 and observed that 13% of the neurons expressed detectable levels of h-αSyn (Figure 2C). On the other hand, antibody mAb1338 against t-αSyn stained all A30P neurons, similarly to non-tg neurons, indicating that the distribution of m-αSyn was similar in both non-tg and tg neurons (Figure 2D). The h-αSyn was expressed mostly in the cell body and around the nucleus of the neurons, but rarely in processes (Figure 2E).

Next, we performed *in situ* PLA between αSyn and the three SNAREs in non-tg and A30P primary neurons. For these analyses, we used mAb1338 antibody, which recognized both m-αSyn and h-αSyn. Theoretically, each PLA dot is the result of the close proximity of one molecule of αSyn and one molecule of one of the SNARE proteins. As a negative control to detect potential unspecific signal, we performed the assay removing one of the primary antibodies, thus one of the secondary antibodies will have no primary antibody to bind and subsequently no PLA signal will be generated.

**Figure 3:**
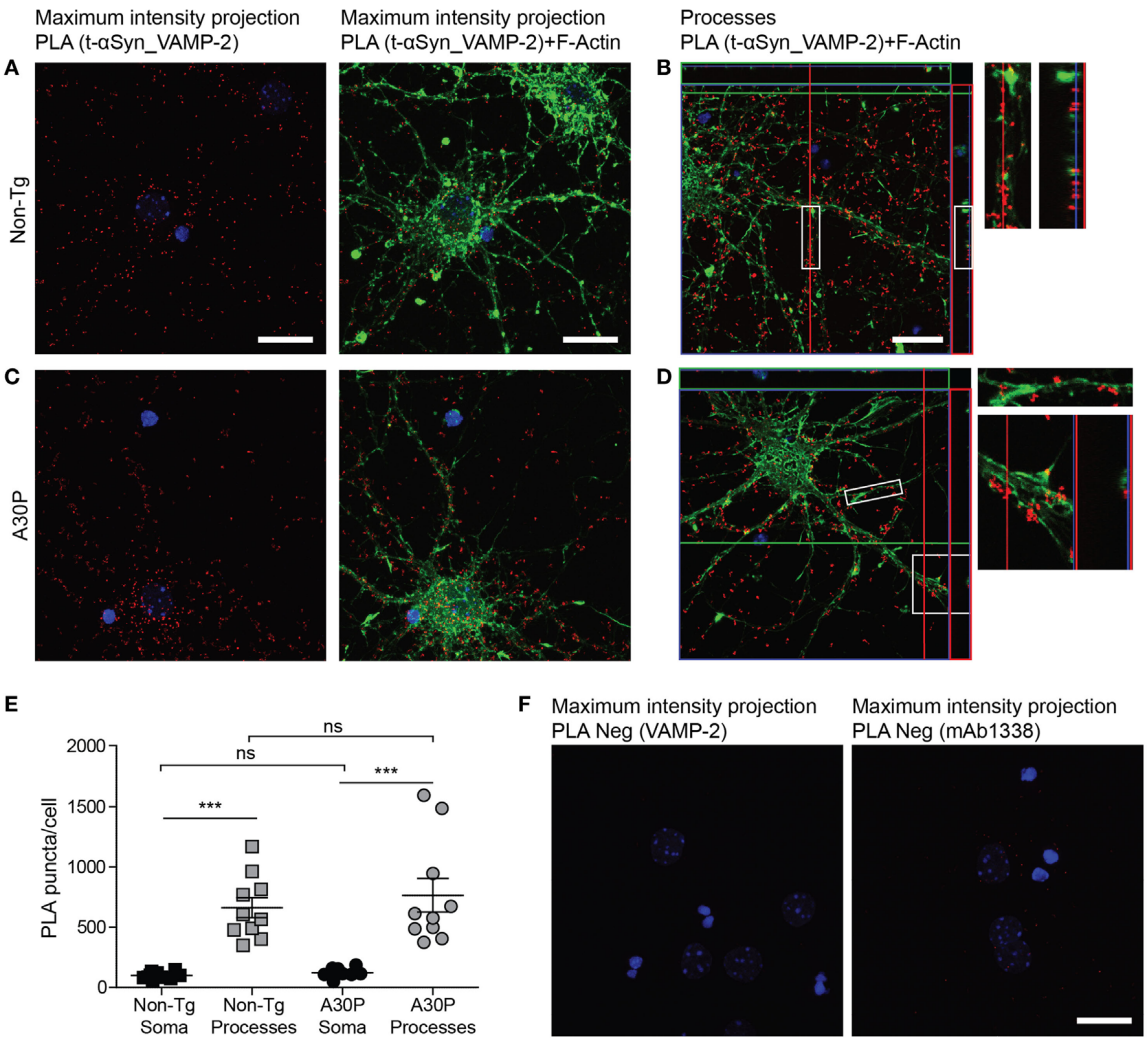
Representative confocal images of *in situ* proximity ligation assay (PLA) between alpha-synuclein (αSyn) (mAb1338) and VAMP-2 (red) in non-tg **(A,B)** and A30P **(C,D)** cortical primary neurons. Maximum intensity projections of a confocal z-stack including a whole cell were performed to observe the maximum amount of PLA puncta **(A,C)**. Cross section of the processes allows visualization of the PLA puncta in an orthogonal view [**(B,D)**, inlet], scale bar 20 µm. F-actin stained by phalloidin-FITC (green) and DAPI (blue). Quantification of amount of PLA puncta per cell in the soma and processes. One-way ANOVA followed by Bonferroni *post hoc* test, non-significant (ns), ***P < 0.001. Error bars represent the SEM **(E)**. Negative control PLA with VAMP-2 antibody only and αSyn antibody (mAb1338) only **(F)**. Scale bar 20 µm.

Abundant PLA signal (red dots) was observed in both sets of primary neurons, indicating a close proximity between αSyn and VAMP-2 (Figures 3A–D). F-actin staining with phalloidin-FITC (green) was performed to observe the cytoskeleton of the neurons and locate the PLA puncta within the cell. Confocal images showed abundant PLA puncta in the processes (Figures 3B,D), a significantly higher number than those found in the soma (Figure 3E). No significant differences were found in the amount of PLA puncta between non-tg and tg A30P αSyn neurons in either processes or the soma (Figure 3E). Very few PLA puncta were observed when the VAMP-2 and αSyn antibodies were used on their own, as negative controls (Figure 3F).

**Figure 4:**
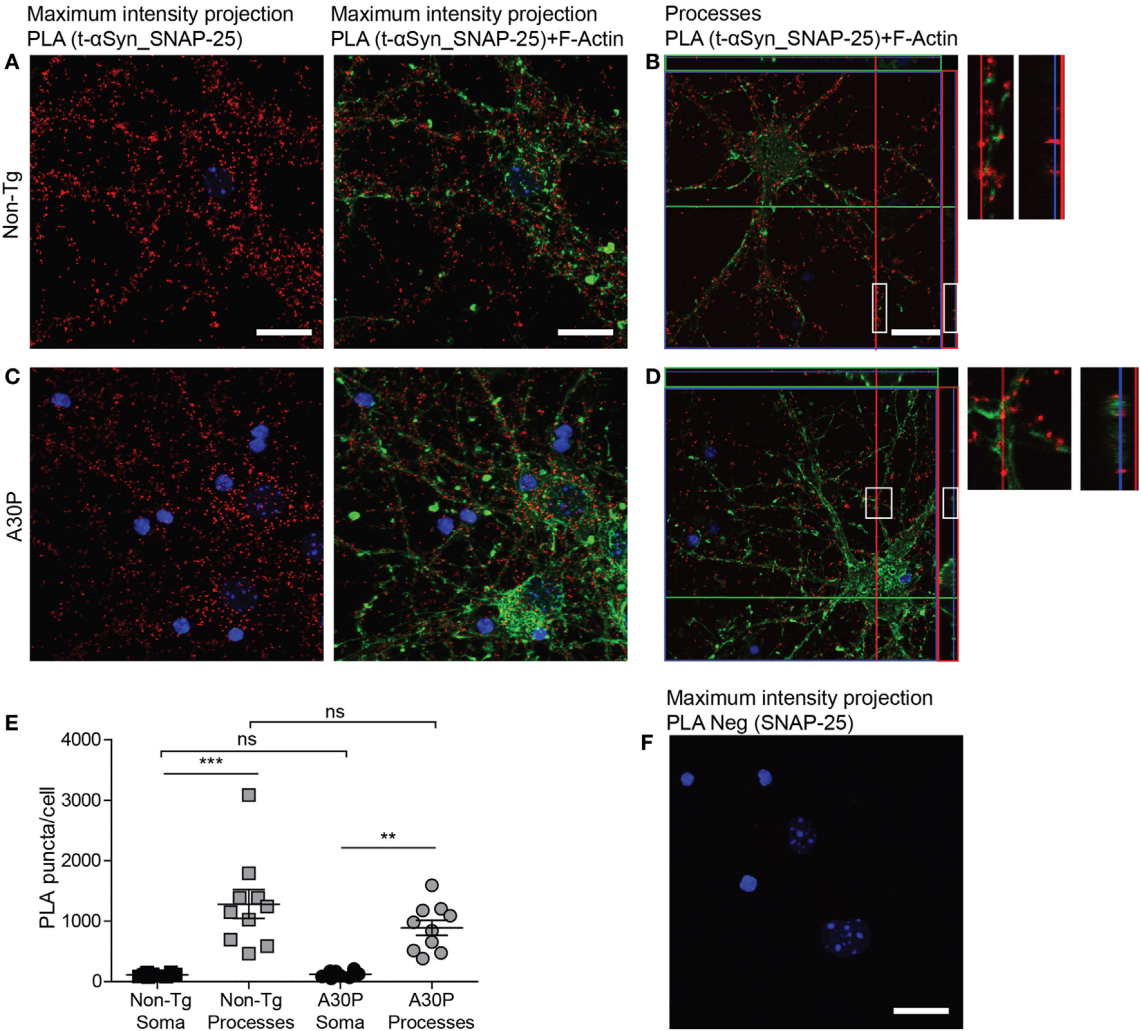
Representative confocal images of *in situ* proximity ligation assay (PLA) between alpha-synuclein (αSyn) (mAb1338) and SNAP-25 (red) in non-tg **(A,B)** and A30P **(C,D)** cortical primary neurons. Maximum intensity projections of a confocal z-stack including a whole cell were performed to observe the maximum amount of PLA puncta **(A,C)**. Cross section of the processes allows visualization of the PLA puncta in an orthogonal view [**(B,D)**, inlet], scale bar 20 µm. F-actin stained by phalloidin-FITC (green) and DAPI (blue). Quantification of amount of PLA puncta per cell in the soma and processes. One-way ANOVA followed by Bonferroni *post hoc* test, non-significant (ns), ***P < 0.001. Error bars represent the SEM **(E)**. Negative control PLA with SNAP-25 antibody only **(F)**. Scale bar 20 µm.

A PLA signal was also observed for αSyn and SNAP-25 (Figures 4A–D), and the distribution of the PLA puncta was similar to the αSyn and VAMP-2 PLA, where the PLA puncta were significantly more abundant in the processes than in the soma (Figure 4E). The amount of PLA puncta per cell was not significantly different between non-tg and tg cells (Figure 4E). Very low signals were observed in control experiments when SNAP-25 antibody was used alone in PLA (Figure 4F).

**Figure 5:**
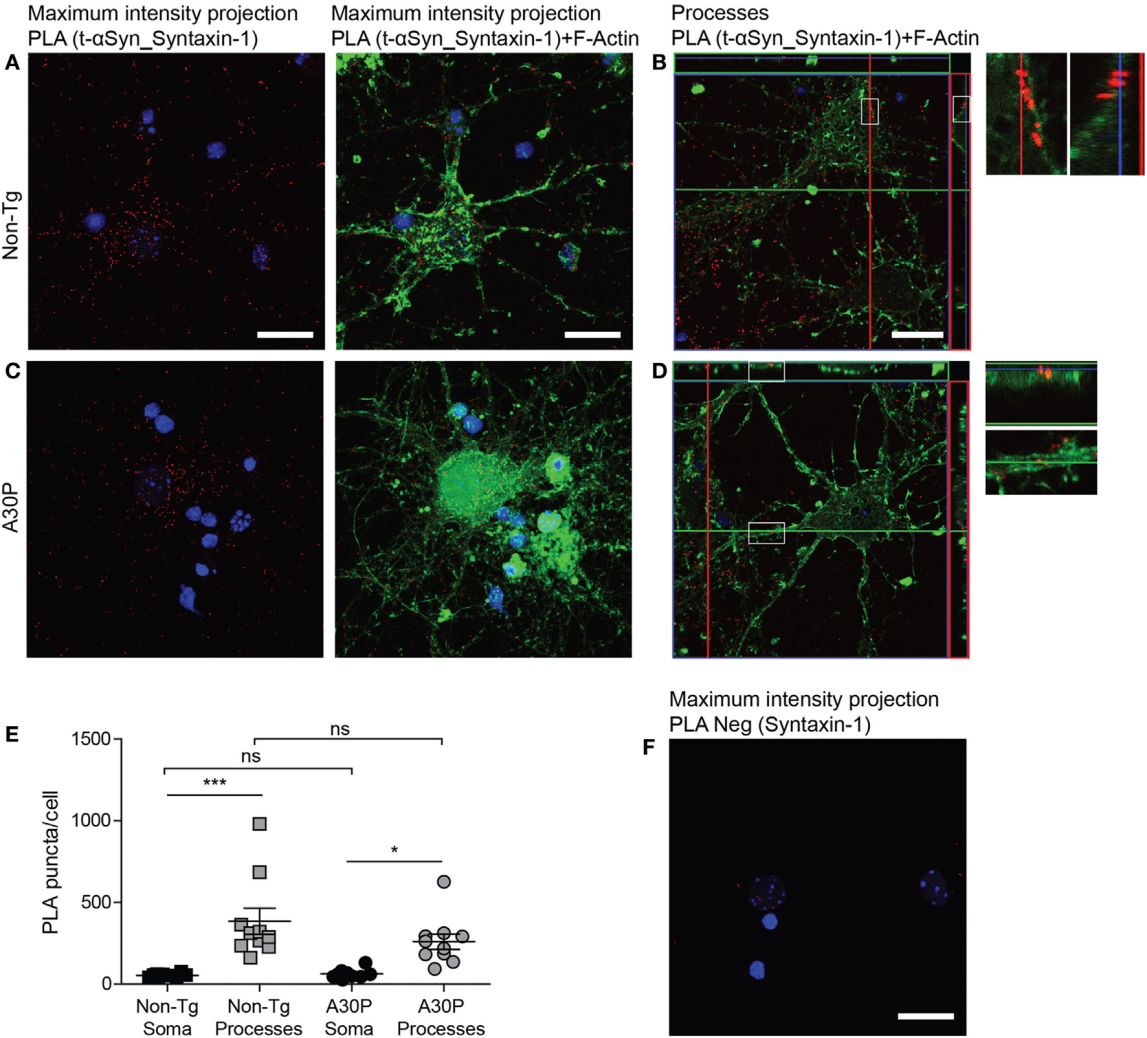
Representative confocal images of *in situ* proximity ligation assay (PLA) between alpha-synuclein (αSyn) (mAb1338) and syntaxin-1 (red) in non-tg **(A,B)** and A30P **(C,D)** cortical primary neurons. Maximum intensity projections of a confocal z-stack including a whole cell were performed to observe the maximum amount of PLA puncta **(A,C)**. Cross section of the processes allows visualization of the PLA puncta in an orthogonal view (**B,D**, inlet), scale bar 20 µm. F-actin stained by phalloidin-FITC (green) and DAPI (blue). Quantification of amount of PLA puncta per cell in the soma and processes. One-way ANOVA followed by Bonferroni *post hoc* test, non-significant (ns), ***P < 0.001. Error bars represent the SEM **(E)** Negative control PLA with syntaxin-1 antibody only **(F)**. Scale bar 50 µm.

Proximity ligation assay against syntaxin-1 and αSyn produced a positive signal in both non-tg and tg neurons (Figures 5A–D), but no differences were observed in the amount of PLA puncta between the two types of cells (Figure 5E). As expected due to the subcellular location of syntaxin-1 (Figure 1D), the puncta were significantly more abundant in the processes, compared with the soma (Figure 5E). Very few puncta were observed when the syntaxin-1 antibody was used alone in control experiments (Figure 5F).

**Figure 6:**
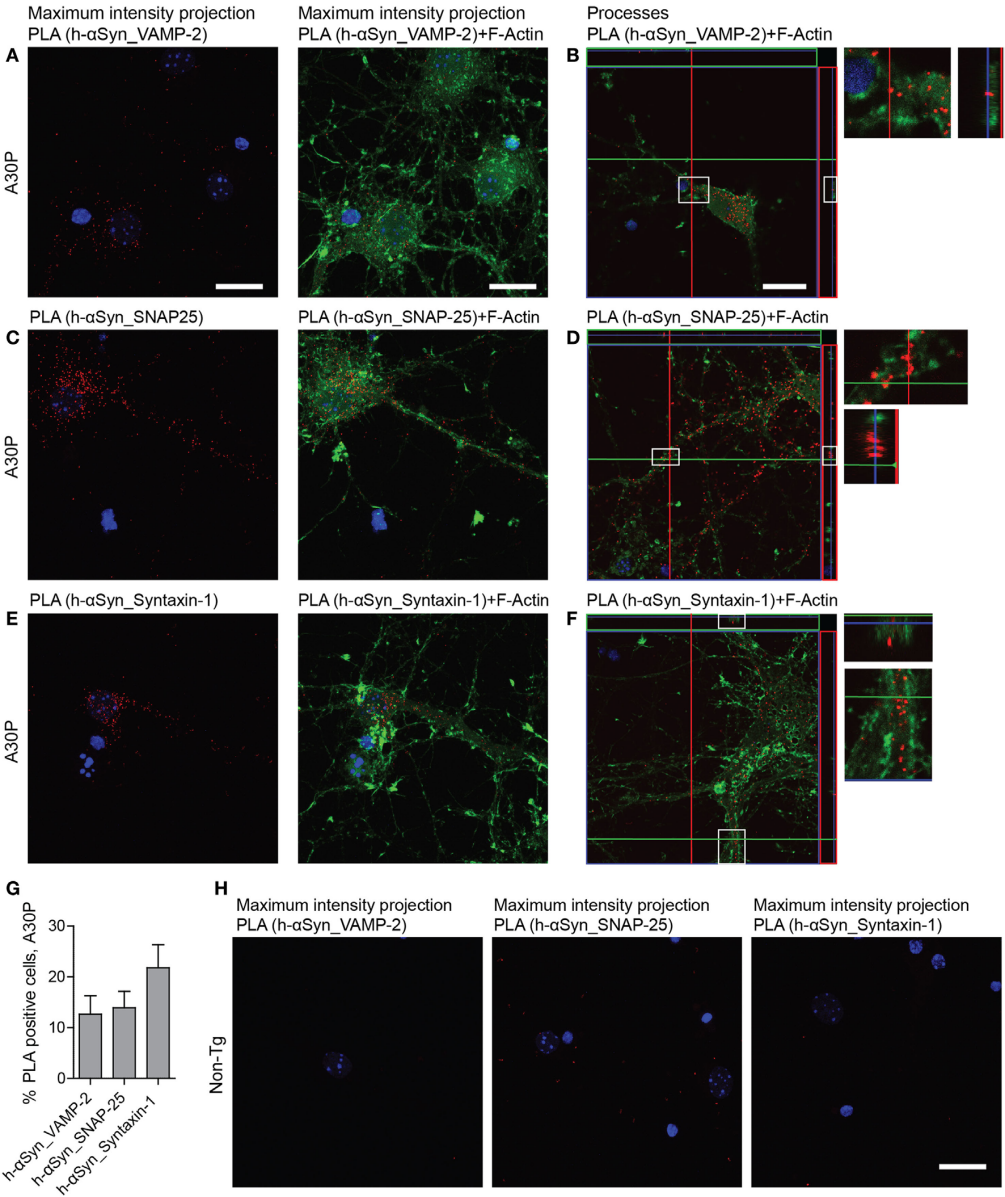
Representative confocal images of *in situ* proximity ligation assay (PLA) (red) between h-alpha-synuclein (αSyn) (Syn 204) and VAMP-2 **(A,B)**, h-αSyn (Syn 204) and SNAP-25 **(C,D)**, and h-αSyn (Syn 204) and syntaxin-1 **(E,F)** in tg A30P neurons and in non-tg neurons **(H)**. Maximum intensity projections of a confocal z-stack including a whole cell were performed to observe the maximum amount of PLA puncta **(A,C,E)**. Cross section of the processes allows visualization of the PLA puncta in an orthogonal view [**(B,D,F)**, inlet], scale bar 20 µm. F-actin stained by phalloidin-FITC (green) and DAPI (blue). Quantification of PLA between h-αSyn and VAMP-2, h-αSyn and SNAP-25, and h-αSyn and syntaxin-1 in A30P neurons **(G)**.

To study the difference in distribution of the interaction between the SNAREs and m-αSyn or h-αSyn, respectively, we performed PLA with the h-αSyn specific antibody Syn 204 and VAMP-2, SNAP-25 and syntaxin-1 (Figures 6A–F). All three antibody combinations showed similar results: only a fraction of the cells were PLA positive, in agreement with what we observed with conventional immunofluorescence with Syn 204, where 13% of all cells expressed h-αSyn (Figure 6G). Most of the h-αSyn-SNARE PLA signal was observed in the neuronal body, with some rare instances where the signal could also be seen in processes. As expected, very few PLA puncta were observed in non-tg primary neurons with h-αSyn specific antibody Syn 204 (Figure 6H).

**Figure 7:**
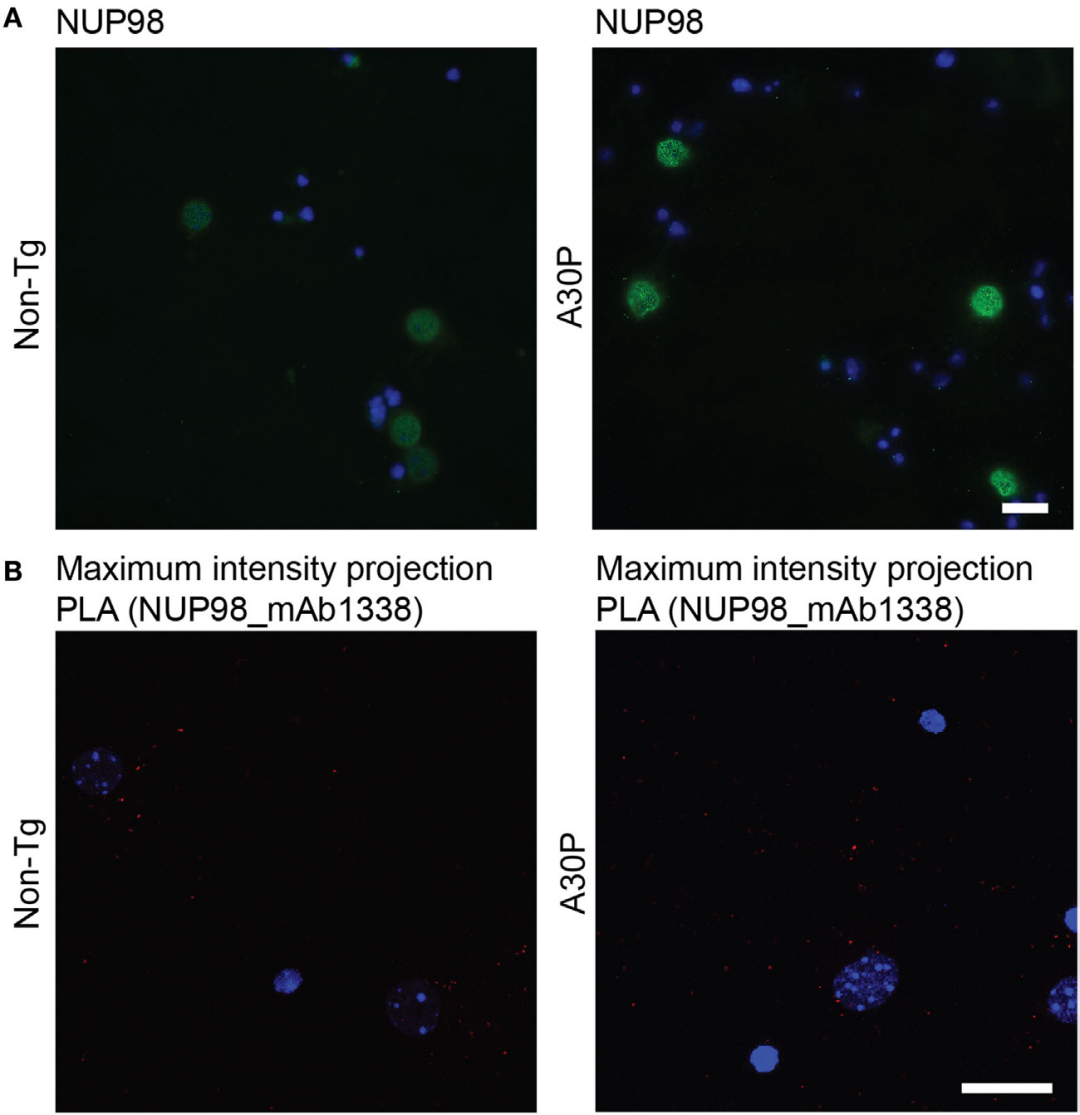
Representative images of NUP98 immunofluorescence staining (green) in non-tg and A30P alpha-synuclein (αSyn) tg primary neurons, DAPI (blue) **(A)**. Representative confocal images (maximum intensity projection) of *in situ* proximity ligation assay (PLA) in non-tg and A30P cortical primary neurons between αSyn (mAb1338) and the NUP98 (red), DAPI (blue) **(B)**. Scale bar 20 µm.

The nuclear pore complex protein NUP98 was used in PLA to further confirm that PLA signal is indicative of proximity between two proteins, as αSyn can be found in the nucleus (18, 19). First we performed immunofluorescence staining of the nuclear pore complex protein NUP98, which gave a perinuclear staining (Figure 7A). PLA with NUP98 and αSyn antibody mAb1338 only produced a few dots, indicating that those two proteins were not in close proximity to each other (Figure 7B). The results of the immunofluorescence staining and PLA were similar in non-tg and tg neurons.

## Discussion

In this study, we can for the first time visualize the co-localization between αSyn and the SNARE proteins in cultured primary neurons by using *in situ* PLA. Previous studies have demonstrated the use of PLA to study αSyn interacting with other proteins, such as the dopamine transporter and αSyn complexes in a tg mouse overexpressing αSyn 1–120 (20). Interactions with TOM20 (21) and the synaptic protein synapsin III (22, 23) have also been observed with PLA. In addition, PLA has been used to specifically detect oligomeric αSyn in brain tissue from PD patients (24).

Direct binding between proteins cannot be proven through *in situ* PLA, as the length of the probes and antibodies theoretically allows proteins with a proximity of up to 40 nm to give a positive PLA signal (25). However, Burré et al. showed that m-αSyn co-immunoprecipitates with VAMP-2, SNAP-25 and syntaxin-1 in brain lysates from non-transgenic mice (7). This observation is indeed in agreement with our PLA results, in which we can see that αSyn is in close proximity to all three SNAREs in non-tg primary neurons, presumably due to its presynaptic localization and membrane binding properties. The PLA puncta were observed to a significantly higher degree in the neuronal processes, presumably indicating the location of synaptic boutons.

We did not observe any significant differences in PLA signal between αSyn and any of the SNARE proteins when comparing primary neurons from non-tg and A30P tg mice. This can be explained by the low amount of h-αSyn present in the tg primary neurons, which according to our ELISA analysis constituted about 2% of the total αSyn detected. This is in contrast to the twofold increase of h-αSyn relative m-αSyn levels observed in adult A30P tg mice (14). The reason for the discrepancy in expressed protein *in vivo* and *in vitro*, as well as for the predominant localization of h-αSyn to the soma, is probably due to the late expression of Thy-1 promoter in the primary neurons (26).

The A30P αSyn mutation exhibits a lower binding affinity for lipid membranes and has a reduced ability to promote SNARE complex formation, compared with wt αSyn and other disease-causing mutations (27, 28). Nevertheless, in the present study we could show that although the amount of h-αSyn positive cells was low (about 13%), positive PLA signals were observed between h-αSyn and all three SNARE proteins. This could perhaps be explained by the finding that there is much smaller difference in relative binding affinity between A30P and wt h-αSyn for highly curved membranes such as synaptic vesicles (6).

Taken together, we can demonstrate that *in situ* PLA is a suitable technique for the study of αSyn co-localization with SNARE proteins in primary neurons. The finding of robust PLA signals between m-αSyn and all three SNARE proteins in the processes, suggests that non-tg primary neurons could be a useful model to study the physiological interaction between αSyn and presynaptic proteins.

## Ethics Statement

This study was carried out in accordance with the EU Directive 2010/63/EU for animal experiments. The protocol was approved by the animal ethics committee of Uppsala, Sweden (C75/13, C92/14).

## Author Contributions

LA-G, EP, AE, and JB designed the experiments. LA-G, EP, and VL performed experiments. LA-G, EP, VL, MI, AE, and JB analyzed data. LA-G, EP, MI, and JB wrote the manuscript.

## Conflict of Interest Statement

The authors declare that the research was conducted in the absence of any commercial or financial relationships that could be construed as a potential conflict of interest.

## Supplementary Material

Figure S1 *In situ* PLA (red) between alpha-synuclein (αSyn) and VAMP-2 **(A)**, αSyn and SNAP-25 **(B)**, and αSyn and syntaxin-1 **(C)**. Sample maximum intensity projections of unprocessed images, which were used for quantification of *in situ* proximity ligation assay (PLA) between αSyn and SNAREs in non-tg and A30P cortical primary neurons [**(A–C**), upper panel]. The lower panels **(A–C)** display the red channel of the same images after deconvolution, and set to the same threshold as was used for the quantification. All PLA puncta from each plane were quantified with ImageJ 3D Objects Counter on deconvolved and thresholded z-stacks. DAPI in blue. Scale bar 20 µm.
