# Supplementary figures and images for: In Situ Proximity Ligation Assay Reveals Co-Localization of Alpha-Synuclein and SNARE Proteins in Murine Primary Neurons
Source: Front Neurol. 2018 Mar 22;9:180. doi: 10.3389/fneur.2018.00180 (PMC5874290; doi:10.3389/fneur.2018.00180)

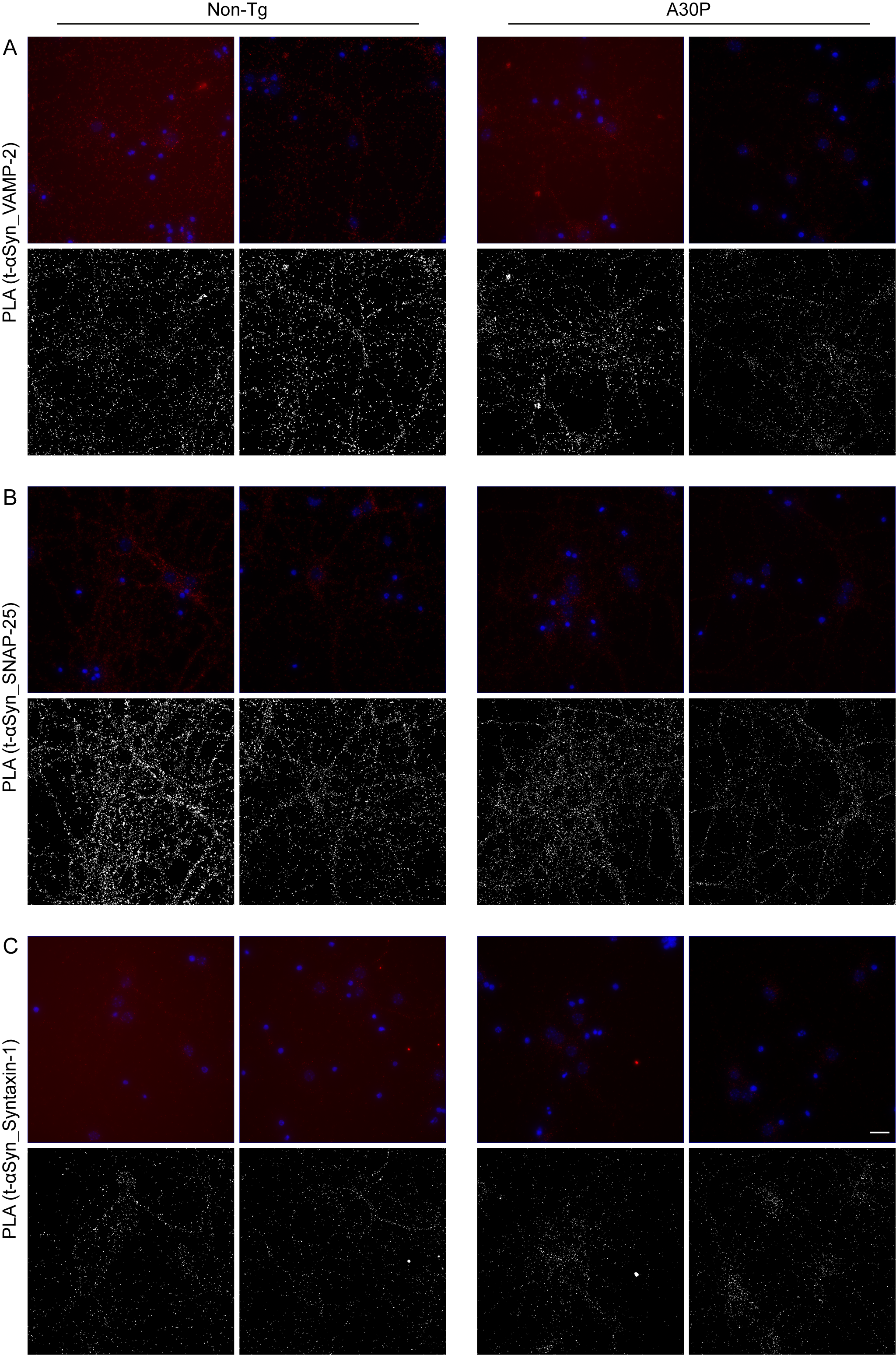

Supplement: Figure S1 — In situ PLA (red) between alpha-synuclein (αSyn) and VAMP-2 (A), αSyn and SNAP-25 (B), and αSyn and syntaxin-1 (C). Sample maximum intensity projections of unprocessed images, which were used for quantification of in situ proximity ligation assay (PLA) between αSyn and SNAREs in non-tg and A30P cortical primary neurons [(A–C), upper panel]. The lower panels (A–C) display the red channel of the same images after deconvolution, and set to the same threshold as was used for the quantification. All PLA puncta from each plane were quantified with ImageJ 3D Objects Counter on deconvolved and thresholded z-stacks. DAPI in blue. Scale bar 20 µm. [file Image_1.TIF]
